# Frequency and reactivity of antigen-specific T cells were concurrently measured through the combination of artificial antigen-presenting cell, MACS and ELISPOT

**DOI:** 10.1038/s41598-017-16549-1

**Published:** 2017-11-27

**Authors:** Chuanlai Shen, Tao Xu, You Wu, Xiaoe Li, Lingzhi Xia, Wei Wang, Khawar Ali Shahzad, Lei Zhang, Xin Wan, Jie Qiu

**Affiliations:** 1 Department of Microbiology and Immunology, Southeast University Medical School, Nanjing, Jiangsu China; 2 Department of Laboratory Medicine, Nanjing KingMed Diagnostics Company Limited, Nanjing, Jiangsu China; 3 Division of Infectious Diseases, Second Hospital of Nanjing, Affiliated Second Hospital of Southeast University, Nanjing, Jiangsu China

## Abstract

Conventional peptide-major histocompatibility complex (pMHC) multimer staining, intracellular cytokine staining, and enzyme-linked immunospot (ELISPOT) assay cannot concurrently determine the frequency and reactivity of antigen-specific T cells (AST) in a single assay. In this report, pMHC multimer, magnetic-activated cell sorting (MACS), and ELISPOT techniques have been integrated into a micro well by coupling pMHC multimers onto cell-sized magnetic beads to characterize AST cell populations in a 96-well microplate which pre-coated with cytokine-capture antibodies. This method, termed AAPC-microplate, allows the enumeration and local cytokine production of AST cells in a single assay without using flow cytometry or fluorescence intensity scanning, thus will be widely applicable. Here, ovalbumin_257–264_-specific CD8^+^ T cells from OT-1 T cell receptor (TCR) transgenic mice were measured. The methodological accuracy, specificity, reproducibility, and sensitivity in enumerating AST cells compared well with conventional pMHC multimer staining. Furthermore, the AAPC-microplate was applied to detect the frequency and reactivity of Hepatitis B virus (HBV) core antigen_18–27_- and surface antigen_183–191_-specific CD8^+^ T cells for the patients, and was compared with conventional method. This method without the need of high-end instruments may facilitate the routine analysis of patient-specific cellular immune response pattern to a given antigen in translational studies.

## Introduction

Antigen-specific T lymphocytes (AST) mediate adaptive immune response; thus, they play crucial roles in health and disease. The enumeration and functional evaluation of diverse T cell populations in an individual’s T cell repertoire may provide a detailed picture of the physiological status, pathological course, and dynamic immune response to certain antigens associated with pathogens, allergens, cancer cells, self-proteins, allograft, or vaccines; thus that might help guide the design of individual-specific immunotherapy. To date, soluble peptide-major histocompatibility complex (pMHC) tetramers and multimers have become the gold-standard tool to define the frequency of AST populations by flow cytometry^1,2^. Cytokine intracellular staining through flow cytometry and enzyme-linked immunospot (ELISPOT) assay have also be widely used to identify the AST cells by detecting the produce of cytokines under the stimulation of antigen or peptides. Furthermore, pMHC multimer staining is combined with phenotypic molecules staining, intracellular cytokine staining or CFSE dilution by using polychromatic flow cytometry to concurrently determine the frequency of AST cells and their activated and memory status, inhibitory receptor expression, cytokine production, degranulation or proliferative capacity in a single assay^3–7^. In addition, pMHC multimer is combined with magnetic-activated cell sorting (MACS) to purify the AST cells through the separation column and followed by polychromatic flow cytometry^8,9^ or ELISPOT assay for the detection of precursor frequencies of naïve AST cells^10^ or adoptive transfer of AST cells^11,12^.

More recently, cellular array-based screening strategies have been developed using microarrays of immobilized pMHC tetramers or dimers^13–19^. By utilizing predetermined spatial coordinates rather than a panel of fluorescent tags in a flow cytometry setting, pMHC microarrays allow the simultaneous identification and characterization of a large number of T cell receptor (TCR) specificities. Deviren fabricated the protein microarrays by spotting the H-2K^b^-Ig dimers loaded with SIYRYYGL peptide onto a film-coated glass surface with a high density to enumerate the carboxyfluorescein succinimidyl ester (CFSE)-labeled 2 C CD8^+^ T cells which mixed with splenocytes from C57BL/6 J mouse^14^. They demonstrate the feasibility of using pMHC microarrays to selectively capture and enumerate antigen-specific CD8^+^ T cells. Furthermore, artificial antigen-presenting microarrays have been established by co-immobilizing pMHC tetramers, costimulatory antibodies, and cytokine-capture antibodies in each spot to screen for ASTs and to detect their local functional responses^15–17^. Although encouraging results and prospects have been reported, there are still challenges for pMHC microarrays. First, unlike pMHC tetramer staining, the purpose of the cellular microarray is to determine (or semi-quantify) the presence of AST cells rather than providing an exact frequency as a result of the indirect readouts by fluorescence intensity scanning or resonance imaging^17,20,21^. Second, in the artificial antigen-presenting arrays, caution must be taken when interpreting the antigenic repertoire from the cytokine response, because only a few AST cells might produce cytokines upon capture^14^. In addition, the spot-to-spot reproducibility, detection limit, and specificity remain to be confirmed and improved. Previously, only a single study has addressed the spot-to-spot reproducibility, which improved by mild shear flow conditions, but without reporting either the within-run or between-run coefficient variation.

In this report, we developed an artificial antigen-presenting cell microplate (termed AAPC-microplate) by co-coupling pMHC multimers and anti-CD28 mAbs onto magnetic beads to sorting and enumerating the AST cells by MACS in a micro well, where cytokine-capturing antibodies were pre-coated, and followed by local cytokine production of AST cells by modified ELISPOT. This method allows the quantification and functional analysis of AST cells in a micro well without the requirement of fluorescence staining and flow cytometry, thus will be widely applicable and may facilitate the routine analysis of patient-specific cellular immune response to a given antigen in clinical samples. The methodological accuracy, specificity, reproducibility, and sensitivity as well as correlation with conventional methods were characterized by measuring ovalbumin (OVA)-specific CD8^+^ T cells from OT-1 TCR transgenic mice. Furthermore, the AAPC-microplate was applied to detect the frequency and reactivity of Hepatitis B virus (HBV) core antigen- and surface antigen-specific CD8^+^ T cells for the patients with chronic HBV infection, and was compared with conventional pMHC multimer staining plus flow cytometry.

## Results

### Preparation of magnetic AAPC-beads and phenotypic analyses

**Figure 1:**
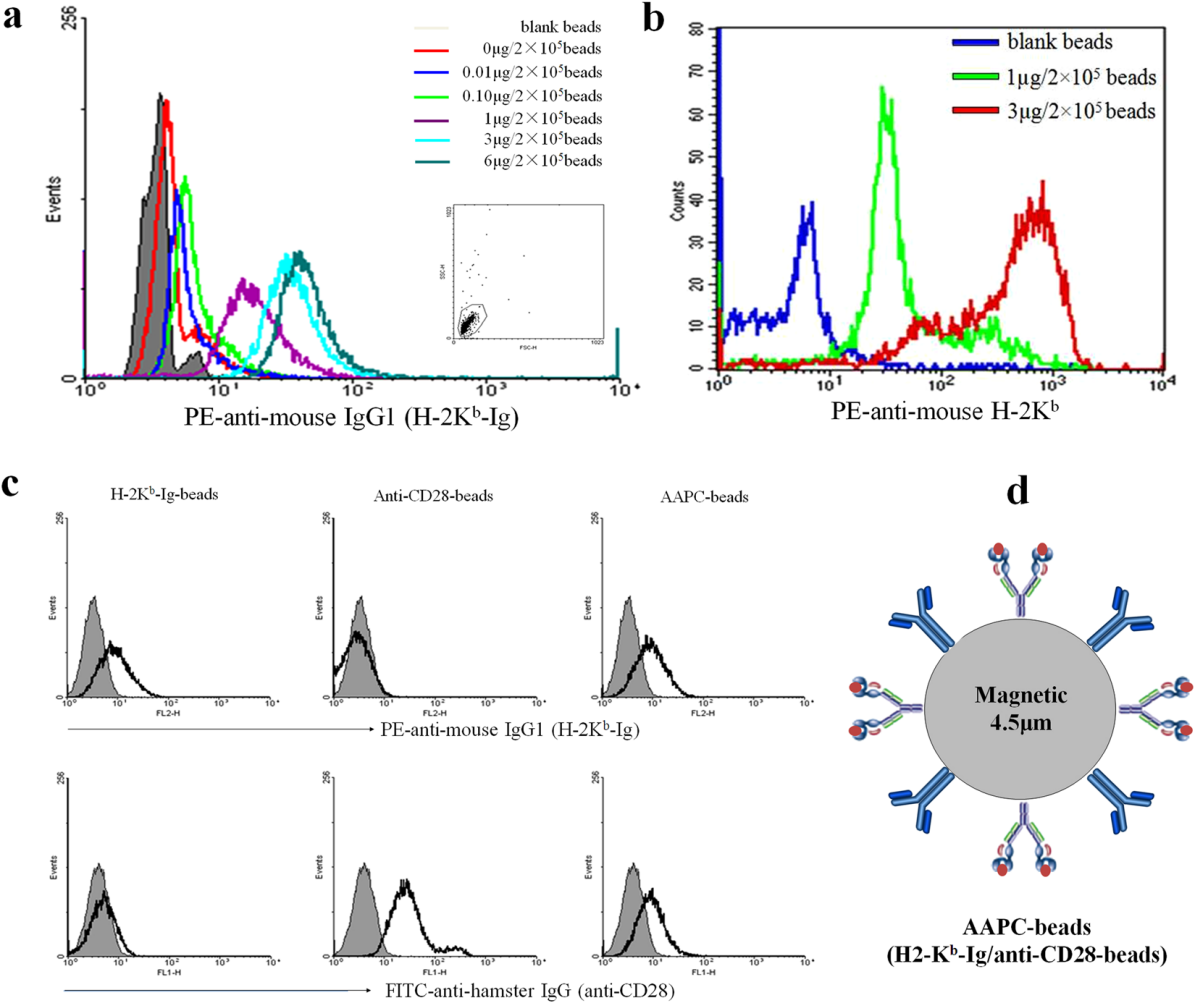
Preparation and phenotypic analyses of AAPC-beads. (**a**) Magnetic Dynal beads were incubated with 1 mL of PBS containing a series of H-2K^b^/OVA_257–264_-Ig dimer quantities at 4 °C overnight with rotation, blocked with BSA, and then stained with PE-labeled anti-mouse IgG1 mAb. (**b**) The magnetic beads were incubated with the indicated amounts of H-2K^b^/OVA_257–264_-Ig dimers overnight followed by BSA blocking first and PE-labeled anti-H-2K^b^ mAb (clone AF6–88.5) staining later. (**c**) Phenotypic analyses of H-2K^b^-Ig-beads, Anti-CD28-beads, and two-signal AAPC-beads (H-2K^b^-Ig/anti-CD28-beads). Gray shaded diagrams represent the isotype control, and solid black lines indicate PE-anti-mouse IgG1 or FITC-anti-hamster IgG staining. (**d)** Schematic representation of AAPC-beads co-coupled with H-2K^b^/OVA_257–264_-Ig dimer and anti-CD28 mAb.

The magnetic Dynabead M-450 Epoxy with a diameter of 4.5 μm is hydrophobic and covered with surface epoxy groups. The epoxide chemistry immobilises ligands containing amino, thiol and hydroxyl functional groups. Here, the Dynabeads were co-coupled with H-2K^b^/peptide-Ig dimers and anti-CD28 mAbs for the preparation of two-signal artificial antigen-presenting cells (AAPC-beads). As shown in Fig. 1a, the beads were able to couple H-2K^b^/peptide-Ig and displayed a significantly increased fluorescence shift of PE at 0.01, 0.1, 1.0, 3.0, and 6.0 μg of H-2K^b^/peptide-Ig per 2 × 10^5^ beads. Figure 1b shows that AAPC-beads were stained effectively with PE-anti-mouse H-2K^b^. Unlike PE-anti-mouse IgG1 that binds to the Ig-Fc domain of the K^b^-Ig dimer, the anti-mouse H-2K^b^ mAb (clone AF6-88.5) recognizes a framework epitope expressed on the β2-microglubulin-associated H-2K^b^ heavy chain^22^. Therefore, the strong binding of PE-labeled anti-mouse H-2K^b^ mAbs with AAPC-beads may imply the correct conformation and appropriate orientation as well as accessibility of H-2K^b^/peptide-Ig dimers onto beads. Figure 1c shows that both H-2K^b^/peptide-Ig and anti-CD28 were immobilized onto the AAPC-beads (Fig. 1d), whereas the anti-CD28-beads and H-2K^b^-Ig-beads only displayed a single signal.

### Enumeration of OVA_257–264_-specific CD8^+^ T cells by AAPC-microplate

**Figure 2:**
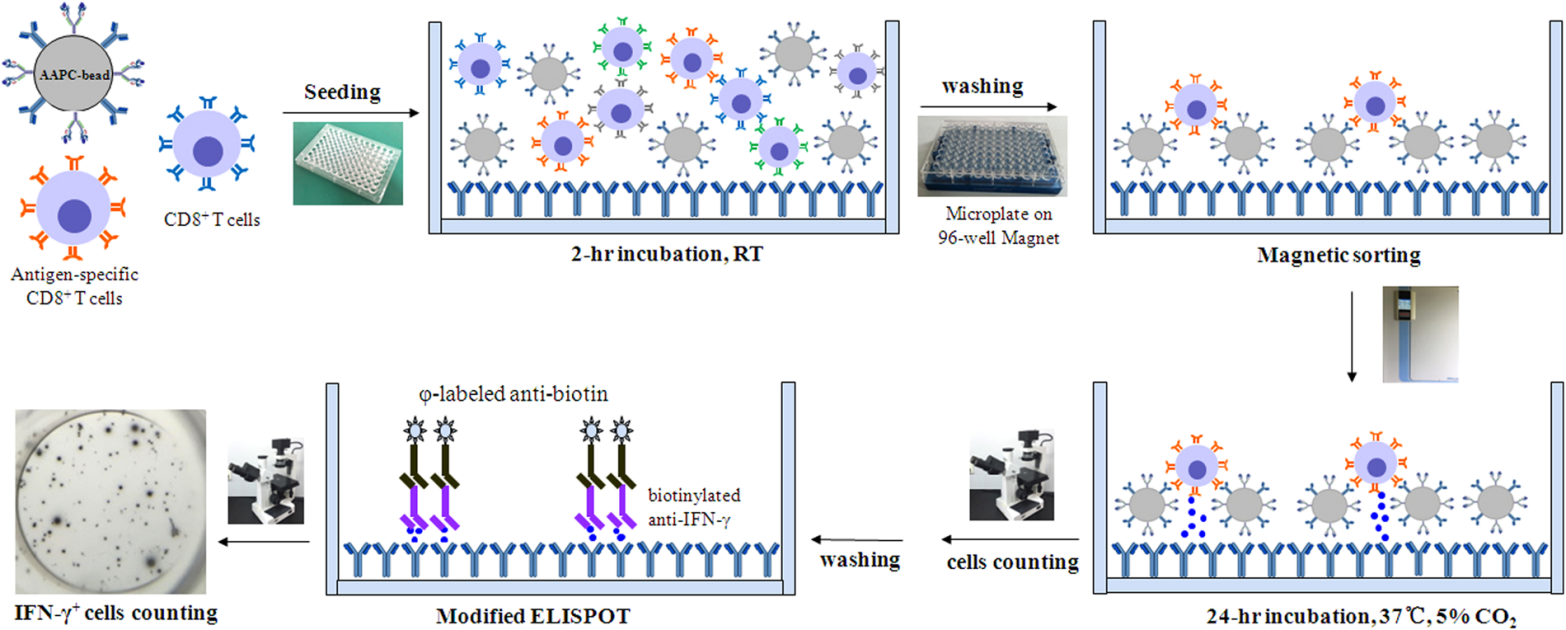
Schematic diagram of the AAPC-microplate experimental procedure.

AAPC-beads, MACS and modified ELISPOT techniques were integrated into a 96-well microplate to develop a new methodological procedure (Fig. 2), termed AAPC-microplate, for the enumeration and local cytokine production of AST cells in a single assay. The methodological accuracy, specificity, reproducibility, and sensitivity in enumerating AST cells was characterized and compared with conventional pMHC multimer staining and flow cytometry by measuring ovalbumin_257–264_-specific CD8^+^ T cells from OT-1 mice.

### Methodological accuracy

**Figure 3:**
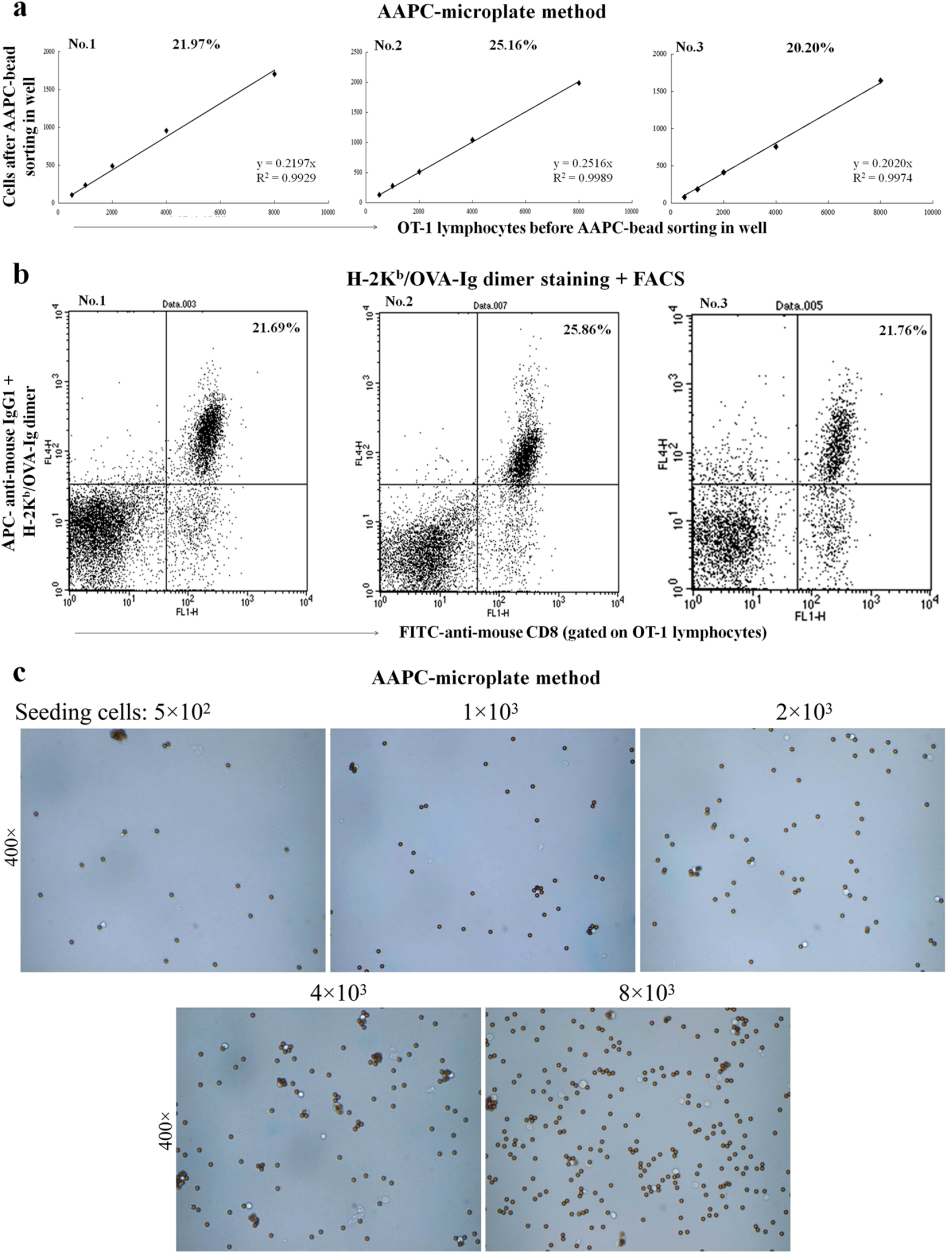
Enumeration of OVA_257–264_-specific CD8^+^ T cells by AAPC-microplate. Spleen cells from individual transgenic OT-1 mouse were detected by the K^b^/OVA-targeted AAPC-microplate method, and were detected in parallel by H-2K^b^-Ig/OVA dimer staining plus flow cytometry. (**a**) The linear relationship between the number of cells retained in micro well before and after AAPC-bead sorting at five cell density groups. The percentage of bead-sorted cells in the microplate was calculated and corrected by a linear regression equation and is shown in the upper part of the diagram. (**b**) Flow cytometric dot plots analyzed by H-2K^b^-Ig/OVA dimer staining. The percentage of dimer^+^/CD8^+^ cells in the gated lymphocyte population is displayed in the top right quadrant. (**c**) Magnetic separation of OVA_257–264_-specific CD8^+^ T cells in AAPC-microplate. The AAPC-beads and cells retained in micro wells were displayed representatively after K^b^/OVA-targeted AAPC-bead sorting and PBS washing at each density group. Magnification is 400 × for each image.

One important procedure was integrated into the experimental protocol to decrease the operating deviation, thus enhancing the methodological accuracy. Lymphocytes were seeded into an AAPC-microplate at five different densities with three replicates for each density. The percentage of bead-sorted cells in each density group was represented by the mean value of three replicate wells. The final percentage of bead-sorted cells was further calculated as the mean ± standard deviation (SD) of five density groups and was corrected using a linear regression equation. Figure 3a,b present the linear regression diagrams of AAPC-microplate method and flow cytometric dot plots of traditional H-2K^b^/OVA-Ig dimer staining, respectively, for the detection of OVA_257–264_-specific CD8^+^ T cells in the lymphocyte populations from three individual OT-1 mice. These data indicate that there is a very good amount-dependent and linear fidelity between the numbers of cells in micro wells before and after AAPC-bead sorting. Figure 3c representatively displays the AAPC-beads and cells retained in micro wells after K^b^/OVA-targeted AAPC-bead sorting and PBS washing at each density group.

**Figure 4:**
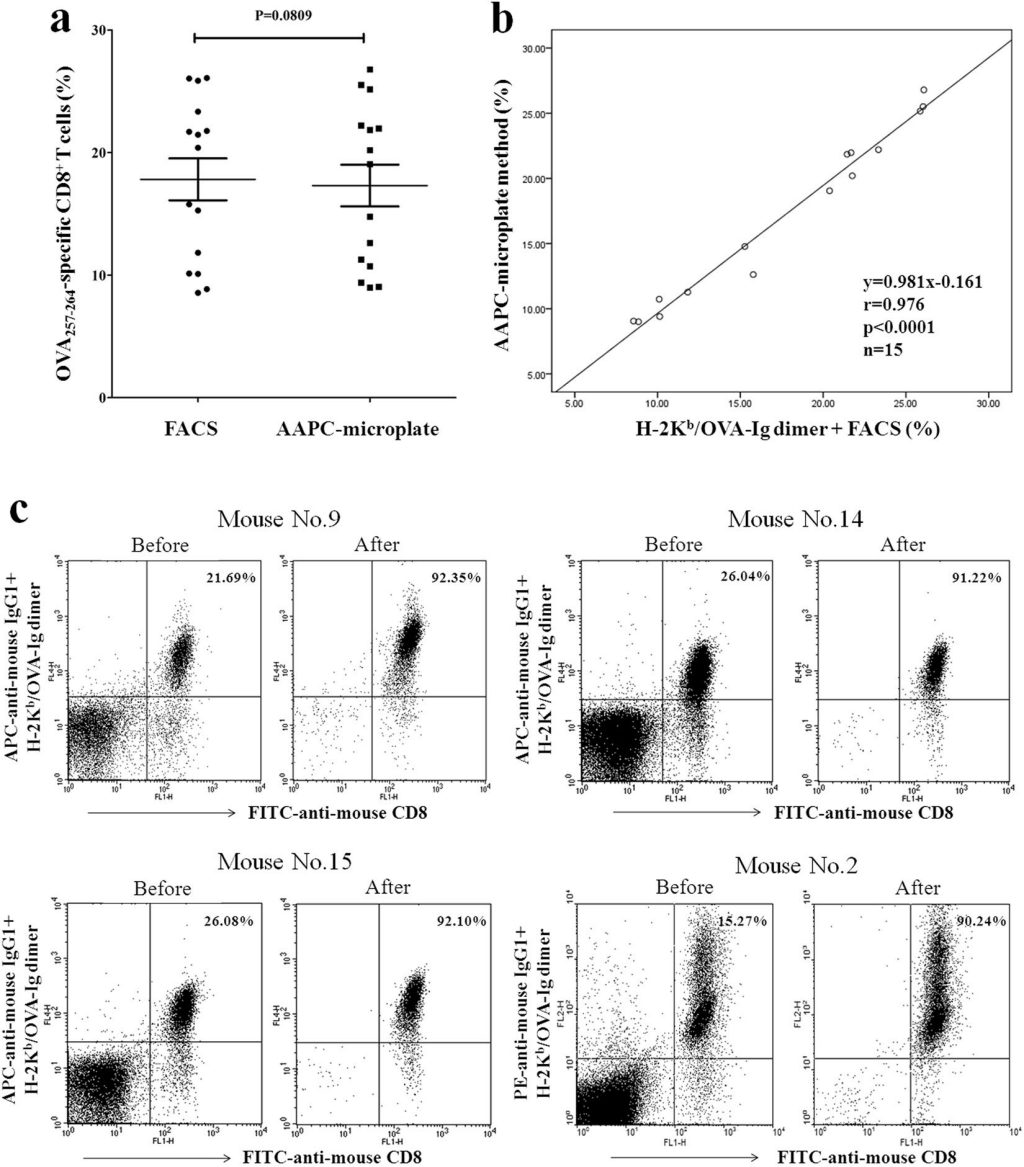
Comparison of the AAPC-microplate method with conventional flow cytometry and the specificity of AAPC-microplate method. Lymphocytes from fifteen individual OT-1 mice were detected by the AAPC-microplate method and traditional H-2K^b^/OVA-Ig dimer staining plus flow cytometry, respectively. (**a**) Paired, two-tailed Student’s t-test for the percentages of OVA_257–264_-specific CD8^+^ T cells detected by the two methods. Data are presented as mean ± SD. (**b**) The correlation coefficient between the AAPC-microplate method and H-2K^b^-Ig/OVA dimer staining as analyzed by two-tailed Pearson correlation. (**c**) Specificity of AAPC-microplate method. Lymphocytes from four individual OT-1 mice were detected by the AAPC-microplate method and traditional H-2K^b^/OVA-Ig dimer staining plus flow cytometry, respectively. Furthermore, the cells retained in each well were harvested after AAPC-bead sorting and stained with H-2K^b^/OVA_257–264_-Ig dimer plus flow cytometry. The percentage of Dimer^+^/CD8^+^ in the retained cells is shown in the top right quadrant as the purity of OVA_257–264_-specific CD8^+^ T cells after AAPC-bead sorting, and compared with the percentage of OVA_257–264_-specific CD8^+^ T cells in lymphocytes before AAPC-bead sorting.

Table S1 shows the percentages of OVA_257–264_-specific CD8^+^ T cells in the lymphocyte populations from fifteen individual OT-1 mice as detected by the AAPC-microplate method and traditional H-2K^b^/OVA-Ig dimer staining. The percentages detected by AAPC-microplate method were comparable with that detected by H-2K^b^/OVA-Ig dimer staining (p = 0.0809, paired, two-tailed Student’s t-test) (Fig. 4a). The correlation coefficient (r value) between the two methods was 0.976 (Fig. 4b) as analyzed by two-tailed Pearson, implying a high degree of concordance between the AAPC-microplate method and the traditional flow cytometry.

### Methodological specificity

The specificity of AAPC-microplate process is mainly dependent upon the specific binding of high-density pMHC multimers onto AAPC-beads with TCRs onto AST cells and the following magnetic separation. The movement or flow of beads in micro wells under mild shaking can increase the encounters between AAPC-beads and AST cells. The magnetic separator for 96-well microplate can produce a more uniform distribution of AAPC-beads and captured T cells during the washing of unbound cells in micro wells. Another crucial point to improve both the specificity and sensitivity of the method is CD8^+^ T cell enrichment prior to AAPC-bead sorting.

Cells were collected from each well after AAPC-bead sorting and stained with H-2K^b^/OVA_257–264_-Ig dimer followed by flow cytometry. As shown in Fig. 4c, the purity of K^b^/OVA dimer^+^/CD8^+^ T cells was increased to 92.35%, 91.22%, 92.10% or 90.24% after AAPC-bead sorting in four individual samples. Few CD4^+^ T cells and non-T cells were present in the flow cytometric spots. These data indicate, to some extent, a high specificity for the AAPC-microplate method. Since the AAPC-microplate process has yet to be optimized, the current specificity indicates the potential for improvement.

### Methodological reproducibility

Fifteen OT-1 mice were detected independently by the AAPC-microplate method for enumerating OVA_257–264_-specific CD8^+^ T cells. The within-run coefficient variation (CV) of five cell density groups (3 wells per density) in each independent test was in the range of 3.11–12.06% (6.51 ± 0.03%) (Table S1). These data represent an acceptable well-to-well reproducibility. Moreover, a cell sample from the same individual OT-1 mouse was detected repeatedly in three independent experiments at different time points by the AAPC-microplate method. As shown in Table S2, three cell samples were tested repeatedly. The between-run CV was 6.88%, 3.22% and 2.40%, respectively. These data imply a reliable reproducibility and precision of the AAPC-microplate method.

### Methodological sensitivity

**Figure 5:**
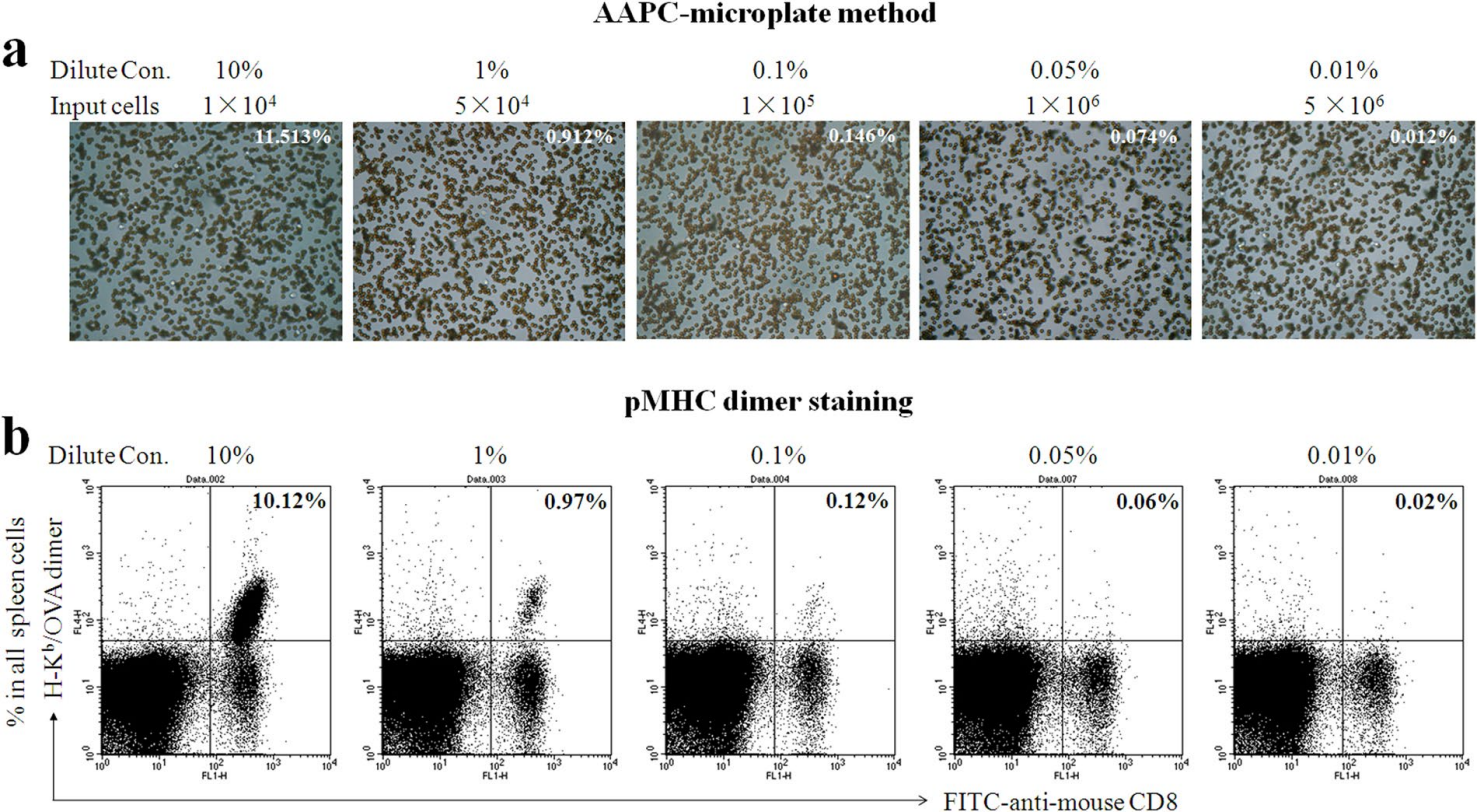
Detection limit of the AAPC-microplate method. OVA_257–264_-specific CD8^+^ T cells from an OT-1 mouse were diluted to 10%, 1%, 0.1%, 0.05%, and 0.01% (Dilute Con.) with splenocytes from a C57BL/6 mouse. Then the diluted lymphocytes were input into micro well at 1 × 10^4^, 5 × 10^4^, 1 × 10^5^, 1 × 10^6^, or 5 × 10^6^ per well, respectively (Input cells), followed by CD8^+^ T cells enrichment, transfer to the micro well in AAPC-microplate and final AAPC-beads sorting in micro well. (**a**) The five diluted cell suspensions were detected by the AAPC-microplate method. The percentage of OVA_257–264_-specific CD8^+^ T cells was calculated by the formula: percentage = cells retained in the micro well after aAPC-beads sorting / lymphocytes input into the micro well before CD8^+^ T cell enrichment, and is shown in the right top of the cell separation image in micro wells. Magnification is 400 × for each image. (**b**) The five diluted cell suspensions were also detected by H-2K^b^/OVA_257–264_-Ig dimer staining plus flow cytometry. The frequency of OVA_257–264_-specific CD8^+^ T cells is shown in the top right quadrant.

In order to enumerate the low-frequency AST cells of interest in clinical sample, an increased number of lymphocytes should be input into micro well or tube for CD8^+^ T cell enrichment first and AAPC-bead sorting in micro well later, thus improving the assay sensitivity and reproducibility. Here, to define the detection limit of AAPC-microplate method, the lymphocytes of OT-1 mouse, which contains high-frequency OVA_257–264_-spcific CD8^+^ T cells as detected by H-2K^b^/OVA_257–264_-Ig dimer staining plus flow cytometry, were diluted to five concentrations containing 10%, 1%, 0.1%, 0.05%, and 0.01% of OVA_257–264_-spcific CD8^+^ T cells (Fig. 5a) in splenocytes from a C57BL/6 mouse, and measured by the AAPC-microplate method. In parallel, H-2K^b^/OVA_257–264_-Ig dimer staining plus flow cytometry was also performed to confirm the percentage of OVA_257–264_-spcific CD8^+^ T cells in the diluted cell populations (Fig. 5b). The detection limit of AAPC-microplate method was 0.01% when 5 × 10^6^ of the diluted lymphocytes containing 0.01% of OVA_257–264_-spcific CD8^+^ T cells were input into micro well for CD8^+^ T cell enrichment. Furthermore, when 1 × 10^7^ of the diluted lymphocytes containing 0.001% of OVA_257–264_-spcific CD8^+^ T cells were input into a tube, not a micro well, for CD8^+^ T cell enrichment, the detection limit of AAPC-microplate method was increased to 0.001% (Data not shown). The high sensitivity is comparable to that obtained by standard state-of-the-art flow cytometry.

### Reactivity evaluation of OVA_257–264_-specific CD8^+^ T cells by AAPC-microplate

**Figure 6:**
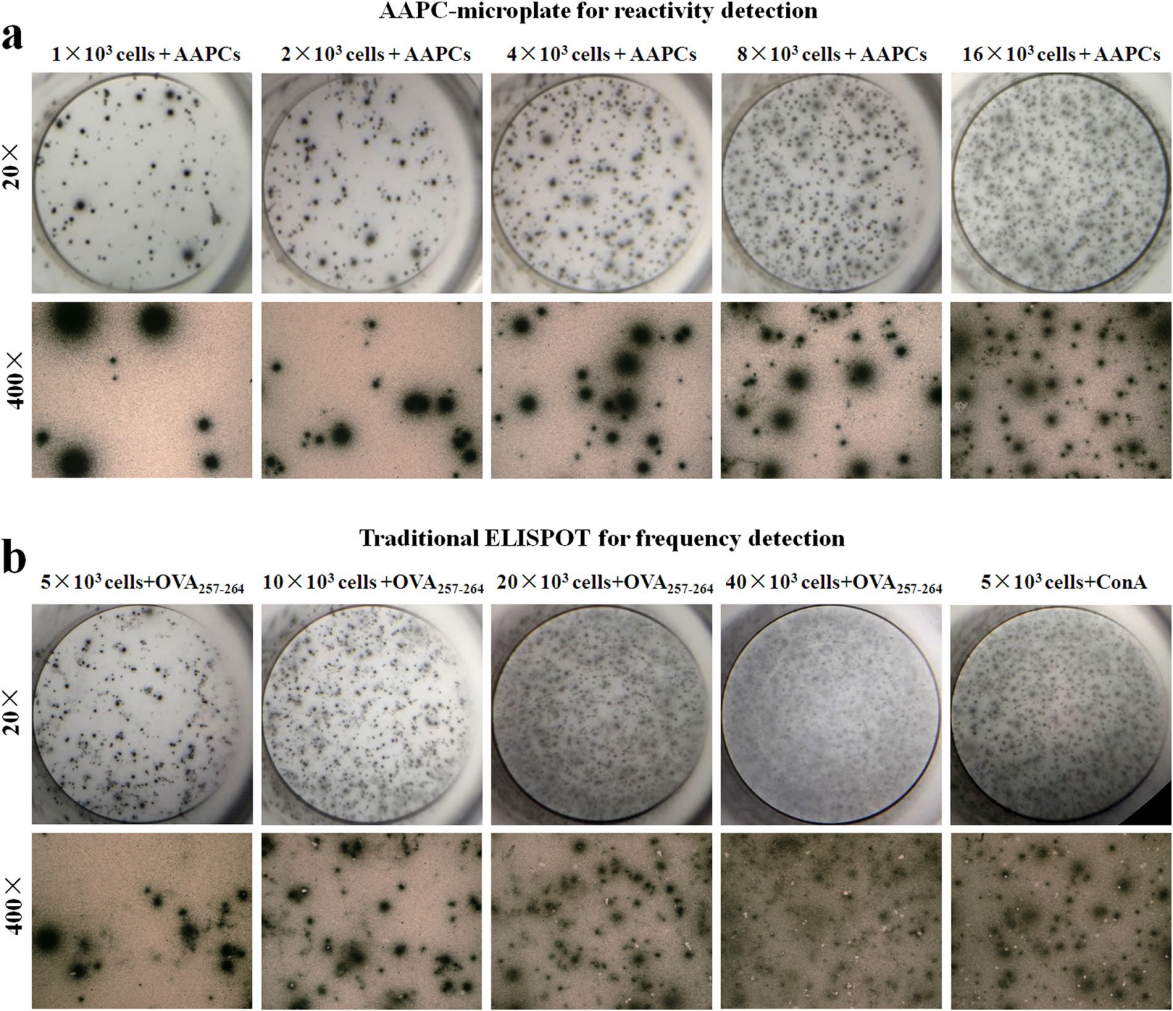
Reactivity detection of OVA_257–264_-specific CD8^+^ T cells by the AAPC-microplate method and traditional ELISPOT assay. After AAPC-bead sorting, the cells retained in each well were further incubated with the AAPC-beads for reactivity evaluation. (**a**) IFN-γ secretion in the OVA_257–264_-specific CD8^+^ T cell population detected by modified ELISPOT after 24-hr stimulation with the two-signal AAPC-beads in the AAPC-microplate. (**b**) IFN-γ secretion in the spleen cells detected by traditional ELISPOT assay after 24-hr stimulation with OVA_257–264_ peptide in a 96-well microplate.

Frequency and reactivity of OVA_257–264_-specific CD8^+^ T cells were detected in a single assay using the AAPC-microplate for eight individual OT-1 mice. After enumerating, cells and AAPC-beads retained in each well were discarded by PBS washing. Consequently, IFN-γ was detected locally with the modified ELISPOT method. As shown in Table S3, the percentage of IFN-γ-secreting cells in the OVA_257–264_-specific CD8^+^ T cell population was in the range of 41.87–49.64% (46.91 ± 2.70%) after 24-hr incubation with AAPC-beads. The representative IFN-γ-positive spots at each cell density were shown in Fig. 6a. These data suggest the feasibility to further functionally evaluate the reactivity of antigen-specific T cells after enumeration in the AAPC-microplate.

### Comparison of the H-2K^b^/OVA dimer staining and AAPC-microplate method with traditional ELISPOT assay

Eight OT-1 mice were detected independently by H-2K^b^/OVA dimer staining, the AAPC-microplate method and traditional ELISPOT assay, simultaneously, for the enumeration of OVA_257–264_-specific CD8^+^ T cells. Figure 6b shows the representative IFN-γ-positive spots at each cell density in traditional ELISPOT assay. As a positive control, the percentage of IFN-γ-secreting cells in the lymphocyte population after ConA incubation was up to 48.46%. As shown in Table S3, in traditional ELISPOT assay, the percentage of IFN-γ-secreting cells in the lymphocyte population after co-culture with OVA_257–264_ peptide was 4.08–14.98%, which was near half of the frequency of OVA_257–264_-specific CD8^+^ T cells detected by H-2K^b^/OVA dimer staining and AAPC-microplate method (Table S3). This difference may owe to the failure of the ELISPOT assay in enumerating non-activated AST cells, because only 46.91 ± 2.70% of OVA_257–264_-specific CD8^+^ T cells produced IFN-γ after stimulation by the two-signal AAPC-beads as detected by the AAPC-microplate.

### Preparation of AAPC-beads for HBV-specific CD8^+^ T cell detection

In order to detect the frequency and reactivity of HBV-specific CD8^+^ T cells using the AAPC-microplate for the clinical samples from patients with chronic HBV infection, the AAPC-beads coupled with HLA-A2/peptide multimers were prepared. Firstly, the recombinant single-chain trimer (SCT) genes of peptide-(GS_4_)_3_-β2m-(GS_4_)_4_-HLA-A2 heavy chain for HLA-A*0201/HBc_18–27_, HLA-A*0201/HBs_183–191_, HLA-A*0203/HBc_18–27_, HLA-A*0203/HBs_183–191_, and HLA-A*0206/HBc_18–27_ complex were successfully constructed and inserted into plasmid pET28a by overlap extension PCR and one-step cloning. Fig. S1a shows the electrophoresis of five DNA fragments amplified in overlap extension PCR for each SCT gene. The SCT proteins were expressed and followed by collection and purification of inclusion bodies (Fig. S1b). Then, the SCT proteins were refolded, concentrated, biotinylated, and coupled onto the cell-sized magnetic beads indirectly by streptavidin. Fig. S1c shows that each type of AAPC-beads could significantly bind with PE-labeled anti-human HLA-ABC (W6/32), a conformation-specific mAb against HLA class I complexes, implying the correct conformational structure and appropriate orientation of HLA-A2/peptide multimers onto beads.

### Enumeration and reactivity evaluation of HBc_18–27_/HBs_183–191_-specific CD8^+^ T cells by AAPC-microplate

**Figure 7:**
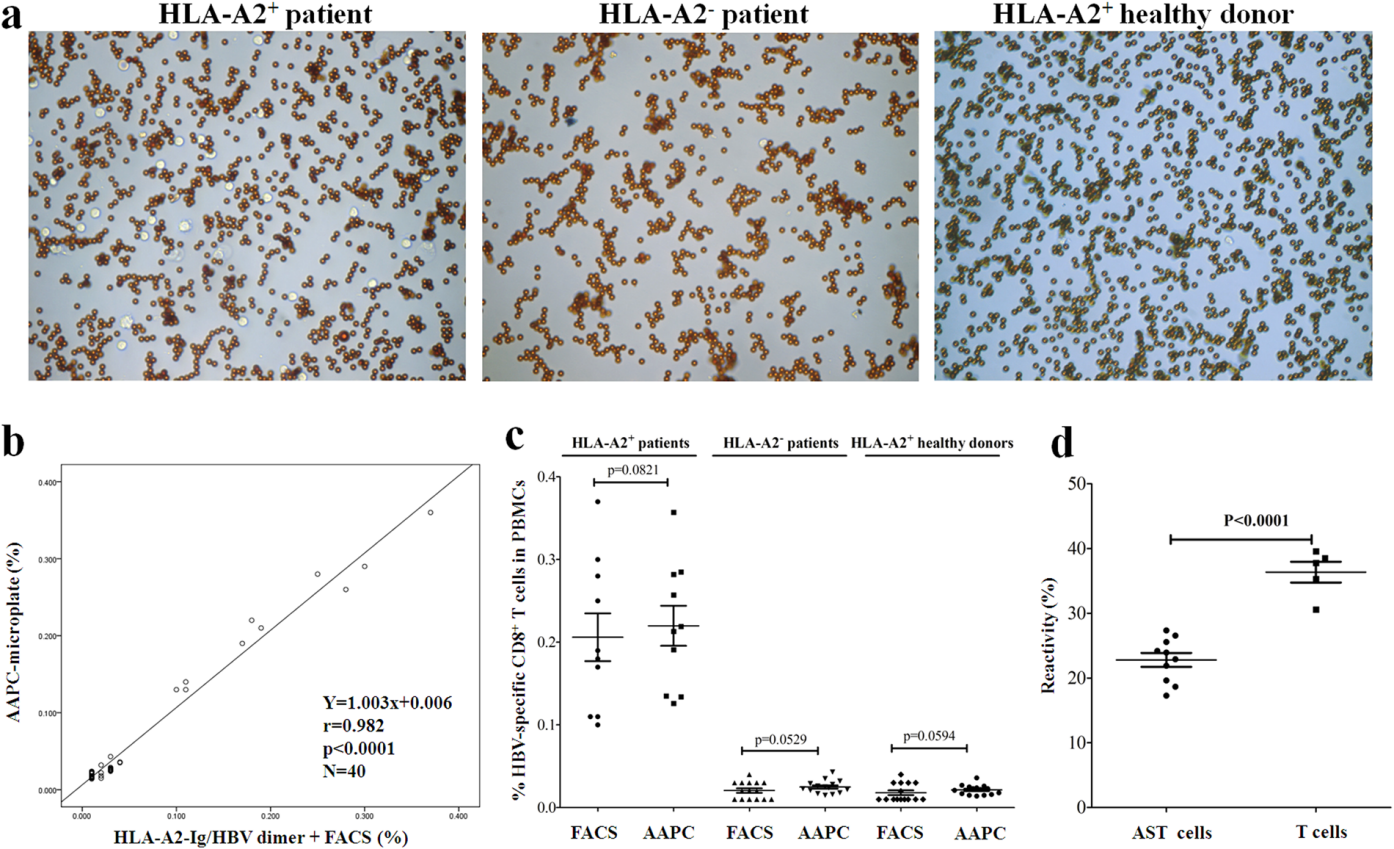
Enumeration and reactivity detection of HBc_18–27_/HBs_183–191_-specific CD8^+^ T cells by AAPC-microplate. PBMCs from all subjects were detected by the AAPC-microplate method and traditional HLA-A2/HBc_18–27_/HBs_183–191_ dimers staining plus flow cytometry. (**a**) Magnetic separation of HBc_18–27_/HBs_183–191_-specific CD8^+^ T cells in the AAPC-microplate. Magnification is 400 × for each image. (**b**) The correlation coefficient between the AAPC-microplate method and flow cytometry as analyzed by two-tailed Pearson correlation. (**c**) The frequencies of HBc_18–27_/HBs_183–191_-specific CD8^+^ T cells in the PBMCs from all subjects as detected by the AAPC-microplate method and flow cytometry (FACS). (**d**) The reactivity of HBc_18–27_/HBs_183–191_-specific CD8^+^ T cells (AST) and whole T cells from the HLA-A2-positve patients with chronic Hepatitis B. HBc_18–27_/HBs_183–191_-specific CD8^+^ T cells retained in micro well after AAPC-bead sorting were further co-incubated with PHA for 24 hrs in the AAPC-microplate. Meanwhile, the PBMC samples from the patients were also co-incubated with PHA for 24 hrs without AAPC-bead sorting. IFN-γ local detection was performed by ELISPOT assay as described and the percentages of IFN-γ-secreting cells in the sorted AST cells and CD3^+^ T cell populations were calculated. Data are presented as mean ± SD.

Blood samples from twenty-five adult inpatients with chronic Hepatitis B and fifteen HLA-A2-positive healthy donors were detected by the AAPC-microplate method and traditional flow cytometry, respectively. The percentages of HBc_18–27_/HBs_183–191_-specific CD8^+^ T cells in the PBMCs from all subjects were shown in Table S4. Figure 7a displays the AAPC-beads and cells in micro wells after HLA-A2/HBV-targeted AAPC-bead sorting for the representative sample of each group. The correlation coefficient (r value) between the two methods was 0.982 as analyzed by two-tailed Pearson (Fig. 7b). No statistical difference was found between the two methods (p > 0.05, paired, two-tailed Student’s t-tests), but HLA-A2-positive patients with chronic Hepatitis B displayed obviously higher frequencies of HBc_18–27_/HBs_183–191_-specific CD8^+^ T cells than the HLA-A2-negative patients or HLA-A2-positive healthy donors, who only presented background level of the AST cells (p < 0.0001, unpaired, two-tailed Student’s t-tests) (Fig. 7c). The flow cytometric dot plots and AAPC-bead sorting picture of each HLA-A2-positive patient with chronic Hepatitis B are presented in Fig. S2.

**Figure 8:**
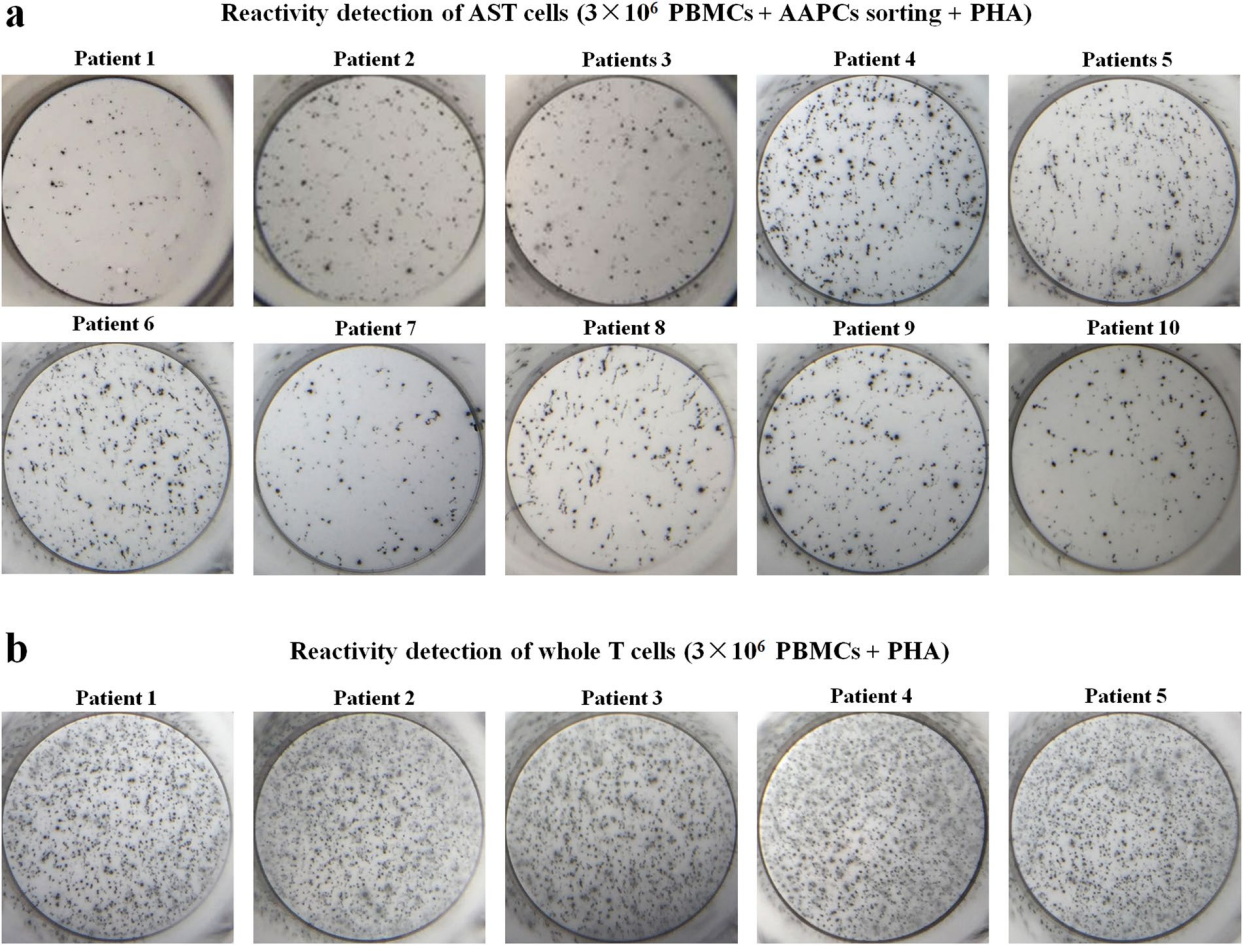
IFN-γ-positive spots in the AST cell population and whole T cell population from the HLA-A2-positive patient with hepatitis B. (**a**) The IFN-γ-positive spots in the AST cells from ten HLA-A2-positive patients as detected by the AAPC-microplate with PHA stimulation. (**b**) The IFN-γ-positive spots in the T cells from five HLA-A2-positive patients as detected by the traditional ELISPOT assay with PHA stimulation.

In the patients with chronic Hepatitis B, the HBV-specific CD8^+^ T cells may have much lower reactivity than the T cells which are not specific for HBV. Therefore, the general reactivity of HBc_18–27_/HBs_183–191_-specific CD8^+^ T cells was further compared with the whole T cell repertoire in the patients. Mitogen PHA rather than the two-signal AAPC-beads was used as stimulator for 24-hr incubation after AAPC-beads sorting, since PHA can activate most of T cell clones in the human T cell repertoire. HBc_18–27_/HBs_183–191_-specific CD8^+^ T cells derived from each HLA-A2-positve patients with chronic Hepatitis B were further co-incubated with PHA (2.5 μg well^−1^) for 24 hrs in the AAPC-microplate. The percentages of IFN-γ-secreting cells in the sorted HBc_18–27_/HBs_183–191_-specific CD8^+^ T cell populations were 22.79 ± 3.40%. Meanwhile, the PBMC samples from the HLA-A2-positive patients with chronic Hepatitis B were also co-incubated with PHA for 24 hrs without AAPC-bead sorting, and followed by IFN-γ local detection as described. The IFN-γ-positive spots in PBMCs were counted and the percentage of IFN-γ-secreting cells in the whole T cell population of PBMCs was calculated, by using the frequency of CD3^+^ T cells in the PBMCs as detected by flow cytometry. As shown in Fig. 7d, the percentages of IFN-γ-secreting cells in the sorted HBc_18–27_/HBs_183–191_-specific CD8^+^ T cell populations were obviously lower than the percentages (36.35 ± 3.58%) in the T cell populations of patients. The IFN-γ-positive spots in AST cell populations (Fig. 8a) and T cell populations (Fig. 8b) from each HLA-A2-positive patient are presented respectively.

## Discussion

Although the similarities to pMHC microarray in concept and applications, the AAPC-microplate method has several points markedly different from the pMHC microarray. Our first emphasis was to use the magnetic beads coupled with pMHC multimers to screening T cells in the AAPC-microplate rather than the uses of pMHC multimers immobilized onto chip in which at least 200 pMHC-TCR interactions are needed for stably capturing and holding a cell against typical flow forces experienced during the washing of unbound cells^4,13^. Magnetic separation has been widely used in cell separation including the selection and expansion of AST cells, thus may enhance the stability of pMHC capturing and holding an AST cell during the washing of unbound cells^8–12,23,24^. Herein, the cell-sized magnetic beads can provide sufficient numbers of pMHC complexes bound to a given cell. Furthermore, the use of smaller magnetic beads should be investigated, because they may facilitate the encounters between beads and AST cells under mild shaking conditions in micro well and improve the specific cell separation under magnetic field^24^.

For pMHC microarrays, the spot size and pMHC density have a major effect on the assay sensitivity. Large spots can improve the detection limit because rare populations of cells can be detected only when a sufficient number of them encounter the appropriate probe region. The reported detection limits as low as 0.1% of total cells on typical microarray are low considering that the endogenous AST cell repertoire is in the range of 0.01–0.1%^13,15,17^. Devirin improved the detection limit to 0.01% by amplifying the spot surface 14 times from typically 0.4 mm in diameter to 1.5 mm in diameter, and by pre-coating the secondary antibody on the microarray surface^14^. The micro well in 96-well AAPC-microplate, a cell culture platform better than microarray slide, provides a much larger surface for AST cell settling than microarray spot; thus, its theoretical detection limit should be much lower than the pMHC microarrays. Here, the rare population as low as 0.01–0.001% was detected well only by inputting more CD8^+^ T cells in micro well. This sensitivity is comparable to that obtained by standard state-of-the-art flow cytometry.

In addition, similar to the pMHC microarray or artificial antigen-presenting array, the AAPC-microplate also allows the detection of multiple AST specificities in a microplate manner. Furthermore, the AAPC-microplate allows the flexible arrays that correspond to the patients’ HLA genotypes because the AAPC-beads coupled with diverse pMHC multimers can be freshly dispensed into microplate in a position-specific manner when use, whereas pMHC microarray had been printed on slide in a pre-arranged format before use. In this study, clinical samples were detected in an array format of diverse samples rather than the diverse AAPC-beads. Actually, we have generated the SCT proteins of 13 HLA-A alleles linked with diverse peptides derived from HBV antigen. The resulting 15 types of HBV-specific AAPC-beads can be dispensed into single 96-well microplate in an array format to detect diverse HBV-specific CD8^+^ T cells for HBV-infected patients with the corresponding HLA-A alleles. However, we cannot find so many commercial HLA-A multimers loaded with indicated HBV antigen epitopes to use as methodological controls. Only HLA-A2-Ig Dimer X can be obtained from Invitrogen. Therefore, we mixed 5 types of AAPC-beads coupled with HLA-A*0201, 0203, and 0206 multimers, which were identified in the HLA-A2-positive patients and donors enrolled in this study, to detect HLA-A2-restricted, HBc_18–27_- and HBs_183–191_-specific CD8^+^ T cells. In parallel, HLA-A2-Ig/HBc_18–27_ dimers and HLA-A2-Ig/HBs_183–191_ dimers were mixed together to detect the same samples by flow cytometry as a conventional method and compared with the AAPC-microplate method.

During the AAPC-microplate process, reliable results depend on several factors, including the amount, functionality, and accessibility of pMHC multimers coupled onto beads, the local cell density and the ratio of cells to AAPC-beads input into micro wells, incubation conditions, plate shaking and washing conditions. Consequently, a standardized operating protocol and a common internal standard of the target cell population in each analysis are required to be developed for routine analysis in translational studies. Here, OVA_257–264_-specific CD8^+^ T cells were used as standard target cells with a high frequency. However, when rare cell population such as clinical samples were tested, the seeding cells should be increased to >1 × 10^6^ cells per well to make sure that more than 100 target cells will be captured by AAPC-beads and retained in well. When less than 0.01% of AST cells were detected, around 1 × 10^7^ cells could be input into a tube, not a micro well, for CD8^+^ T cell enrichment first and then transfer into AAPC-microplate for following enumeration and functional evaluation.

Of course, magnetic sorting of AST cells in microplate with pMHC multimer, MACS, or ELISPOT assay is not new technique. But in the AAPC-microplate procedure, two points are different from the previous researches. Firstly, the two-signal magnetic AAPC beads coupling the antigen signal and co-stimulatory signal were used to purify AST cells in MACS and activate AST cells in micro well followed by ELISPOT for local cytokine detection. To our knowledge, this integration was initially reported here. Secondly, the AAPC-microplate assay for the concurrent quantification and functional evaluation of AST cells does not need the high-end instruments which are required in the pMHC multimer staining combined with intracellular cytokine staining. Here, AST cells and cytokine-secreting cells are counted mainly by the naked eye under an optical microscope. Only when a lot of cells retained in the micro well after AAPC-bead sorting, the automated cell counter is required. Actually, the AST cell of interest is rare cell population in most clinical blood samples, thus the cells or spots retained in the micro well are less than several hundreds which can be counted manually and easily. This may facilitate its wide use at small and medium-sized clinical laboratories, especially in developing countries. Furthermore, high-end automatic readout methods can also be integrated into this system to meet with the demand from large-sized laboratories, such as cell counting using inverted microscope together with spot camera and image quantifying software, or DiO/Calcein labeling and fluorescence scanning, termed on-chip imaging cytometry^25^.

In summary, we developed an AAPC-microplate which combines the antigen-specific features of pMHC multimer, cell separation offered by MACS, co-stimulation of anti-CD28 provided by AAPC-beads, and cellular functional evaluation presented by ELISPOT. Our results demonstrate the feasibility of using AAPC-microplate to enumerate and functionally evaluate multiple AST cells in a single assay. The high accuracy, specificity, reproducibility, and sensitivity compares well with conventional methods. These features, together with no requirement for expensive instruments, suggest its potential for the routine analysis of patient-specific immune response pattern to certain antigen in translational studies.

## Materials and Methods

### Reagents and instruments

Magnetic Dynabeads M-450 Epoxy (Cat. No 14011), Dynabeads Untouched Mouse CD8 Cells Kit, and Dynabeads Untouched Human CD8 T Cells Kit were from Invitrogen (Carlsbad, CA, USA). H-2K^b^-Ig Dimer X, human leukocyte antigen HLA-A2-Ig Dimer X, purified rat anti-mouse CD16/CD32, Human FcR Blocking Reagent, R-phycoerythrin (PE)-mouse anti-mouse H-2K^b^ monoclonal antibody (mAb), Allophycocyanin (APC)-labeled, PE-labeled or purified rat anti-mouse IgG1, fluorescein-5-isothiocyanate (FITC)-mouse anti- hamster IgG, PE-mouse anti-human HLA-A2 (BB7.2), FITC-anti-human CD8 and PE-anti-human CD3 mAbs were purchased from BD Biosciences (Franklin Lakes, NJ, USA). Purified hamster anti-mouse CD28 mAb and mouse anti-human CD28 mAb (Functional grade), PE-anti-mouse CD4 and FITC-anti-mouse CD8a, APC-anti-mouse CD3e and PE-anti-mouse Vα2 TCR, and PE-mouse anti-human HLA-ABC mAb (W6/32) were from eBioscience (San Diego, CA, USA). Mouse interferon-γ (IFN-γ) ELISPOT kit (CT317-T2) and human IFN-γ ELISPOT kit (CT230-T2) were from U-cytech Biosciences (Utrecht, Netherlands). These kits contain the 96-well transparent polystyrene ELISPOT plates which produced by ANIARA Company (Cat. No: ACT350; West Chester, OH). Concanavalin A (ConA) was from Sigma-Aldrich (St. Louis, MO, USA). Serum-free cell culture medium and phytohaemagglutinin (PHA) were from Dakewe Biotech (Shenzhen, China). Lifesep 96 F cell separator was from Dexter Magnetic Technologies, Inc (Eik Grove Village, IL, USA). Automated cell counter (Countstar IC1000) was from Ruiyu Biotech (Shanghai, China). OVA_257–264_ (SIINFEKL), Tyrosinase-related protein 2 (TRP2)_180–188_ (SVYDFFVWL), HBV core antigen (HBc)_18–27_ (FLPSDFFPSV), and HBV surface antigen (HBs)_183–191_ (FLLTRILTI) peptides were synthesized by China Peptides Co. (Shanghai, China), and the purity of each peptide was >95%.

### Mice

C57BL/6 J mice were purchased from the Comparative Medicine Center of Yangzhou University (Yangzhou, Jiangsu, China). Transgenic OT-1 mice were kindly gifted by Dr. Hua Tang (Tai Shan Medical College, Shandong, China) and bred in-house. Mice were maintained in the specific pathogen-free Laboratory Animal Centre of Southeast University (Nanjing, Jiangsu, China), and were used in experiments at 8–12 weeks of age. Animal welfare and experimental procedures were performed in accordance with the Guide for the Care and Use of Laboratory Animals (Ministry of Science and Technology of China, 2006) and were approved by the Animal Ethics Committee of Southeast University.

### Preparation of AAPC-beads for OVA-specific T cell detection

The magnetic Dynabeads (M-450 Epoxy) (2 × 10^5^) were co-incubated with 0.5 μg of rat anti-mouse IgG1 antibody and 1.0 μg of hamster anti-mouse CD28 antibody in sterile 0.1 M phosphate buffered saline (PBS) overnight with rotation at 4 °C, and followed by blocking with 30% (w/v) bovine serum albumin (BSA) in PBS for 3 hrs at 4 °C. Then the beads were coated with 1.0 μg of H-2K^b^/OVA_257–264_-Ig dimer or H-2K^b^/TRP2_180–188_-Ig dimer (non-cognate antigen control) for 2 hrs at 4 °C. After washing twice with PBS, the resulting AAPC-beads were stored in sterile 0.1 M PBS at 4 °C. The control beads (anti-CD28-beads) were also prepared as described in the absence of rat anti-mouse IgG1 antibody and H-2K^b^/peptide-Ig dimer.

To analyze the phenotype of AAPC-beads, all beads were stained with PE-labeled mouse anti-mouse H-2K^b^ mAb or PE-conjugated rat anti-mouse IgG1 (binding to H-2K^b^-Ig dimer) and FITC-anti-hamster IgG (binding to anti-CD28), respectively, for 30 min at 4 °C in the dark, acquired on a FACS Calibur flow cytometer (BD Biosciences, San Diego, CA, USA), and analyzed with WinMDI 2.9 (Purdue University, West Lafayette, IN, USA) or CellQuest software (BD, San Diego, CA, USA).

### Enumeration and reactivity evaluation of OVA_257–264_-specific CD8^+^ T cells by AAPC-microplate

Figure 2 displays the experimental procedure. Firstly, CD8^+^ T cells were separated using a Dynabeads untouched mouse CD8^+^ T cells selection kit. Briefly, lymphocytes enriched from the spleen of OT-1 mice were blocked with anti-mouse CD16/32, then, 5 × 10^7^ cells were incubated with Antibodies Mixture for 20 min at 4 °C. After washing, cells were mixed with Mouse Depletion Dynabeads (5 × 10^7^ beads), and seeded into 96-well round-bottom plates at a density of 1 × 10^3^, 2 × 10^3^, 4 × 10^3^, 8 × 10^3^ and 1.6 × 10^4^ cells per well. Three replicate wells for each density were used. Plates were incubated for 30 min at room temperature (RT) on a mild shaker, and then placed on a 96-well microplate Magnet for 5 min to gather the Dynabeads and CD8^-^ cells onto each well. Supernatant containing CD8^+^ T cells in each well was transferred into a transparent 96-well flat-bottom plate (ANIARA Company) which pre-coated with interferon (IFN)-γ-capturing antibody.

Secondly, AAPC-beads were seeded into the microplate containing CD8^+^ T cells and co-incubated for 2 hrs on a mild shaker at RT. The ratio of AAPC-beads to lymphocytes that were seeded into each well prior to CD8^+^ T cell separation was 1:1. Then, the plate was gently washed three times using serum-free cell culture medium on a 96-well microplate Magnet to discard the cells unbound to AAPC-beads. The cells and AAPC-beads retaining in each well were maintained in serum-free cell culture medium and further incubated for 24 hrs in a humidified incubator with 5% CO_2_ at 37 °C. Then, the cells in each well were counted. When only several hundreds of cells retained in the micro well, manual cell counting was performed independently by the same three investigators under an optical microscope. Only one knows the experimental groups. The other two investigators are blind to the experimental design. The final data was recorded as the mean number rounded to the nearest integer. In case of large discrepancies, a consensus was reached upon joint evaluation. But when a lot of cells retained in the micro well, the automated cell counter (Countstar IC1000, Ruiyu Biotech) was used. The AAPC-microplate was placed on the 96-well plate Magnet for 5 minutes to fix the AAPC-beads on the bottom of each well, and then 20 μl of cell suspension from each well was loaded to the automated cell counter for the rapid cell counting. The frequency of cells sorted by AAPC-beads in lymphocyte populations was finally calculated using a linear regression equation.

After cell counting, the cells and AAPC-beads in each well were discarded by thoroughly washing with PBS. Plate was further incubated for 1 hr at 37 °C with 100 μL of biotin-labeled anti-mouse IFN-γ in each well and followed by additional 1 hr incubation with 50 μL of φ-labeled goat anti-biotin antibodies (gold-labeled anti-biotin antibodies, GABA). Subsequently, the plate was washed thoroughly and incubated with activator I/II solution (silver salt solution, 35 μL well^−1^) for 25–30 min at RT followed by ddH_2_O washing. Finally, the spots appeared in each well and were counted. The percentage of IFN-γ-secreting cells in the OVA_257–264_ antigen-specific CD8^+^ T cell population was calculated and corrected using a linear regression equation.

### Enumeration of OVA_257–264_-specific CD8^+^ T cells by traditional flow cytometry and ELISPOT assay

Lymphocytes isolated from spleen of OT-1 mice were stained with FITC-labeled anti-mouse CD8a, APC-labeled anti-mouse CD3e and PE-labeled anti-mouse TCR Vα2 for 30 min at 4 °C in the dark. For H-2K^b^/OVA_257–264_-Ig dimer staining, H-2K^b^/OVA-Ig dimer or H-2K^b^/TRP2-Ig dimer was first mixed with PE-labeled anti-mouse IgG1 for 30 min at RT. Then, lymphocytes were blocked with anti-mouse CD16/CD32 for 1 hr and incubated with the mixture of H-2K^b^/peptide-Ig dimer and PE-labeled anti-mouse IgG1 for 1 hr at 4 °C. After washing, FITC-labeled anti-mouse CD8a and APC-labeled anti-mouse CD3e were added for additional 30 min incubation. Finally, cells were acquired on a FACS Calibur flow cytometer and analyzed with WinMDI 2.9 or CellQuest.

A mouse IFN-γ ELISPOT kit was used according to the manufacturer’s protocol with modifications. Briefly, a transparent 96-well flat microplate was coated with IFN-γ-capturing antibody and blocked with a non-cognate protein. Lymphocytes were then seeded into the plate at a density of 1 × 10^3^, 2 × 10^3^, 4 × 10^3^, 8 × 10^3^, and 1.6 × 10^4^ cells per well. Three replicates for each density were produced. Cells were maintained in serum-free cell culture medium and incubated with OVA_257–264_ peptide (10 μg well^−1^) or ConA (5 μg well^−1^, positive control) for 24 hrs in a humidified incubator providing 5% CO_2_ at 37 °C.

### Preparation of AAPC-beads for HBV-specific T cell detection

HLA-A*0201/HBc_18–27_, HLA-A*0201/HBs_183–191_, HLA-A*0203/HBc_18–27_, HLA-A*0203/ HBs_183–191_, and HLA-A*0206/HBc_18–27_ complexes were generated as our previous report^26^. Briefly, the single-chain trimer (SCT) gene of peptide-(GS_4_)_3_-β2m-(GS_4_)_4_-HLA-A2 heavy chain was constructed and inserted into plasmid pET28a by overlap extension PCR and one-step cloning (Fig. S3). The SCT protein was expressed in *Escherichia coli*, then purified using Ni^+^-chelating affinity column, refolded by dilution refolding method, and biotinylated in the presence of BirA and d-biotin.

2 × 10^5^ magnetic beads were incubated with 1.0 μg of streptavidin and 1.0 μg of mouse anti-human CD28 overnight with rotation at 4 °C and followed by blocking with 30% BSA in PBS for 3 hrs at 4 °C. After washing, the beads were further incubated overnight with rotation at 4 °C with 4.0 μg of each type of biotinylated HLA-A2/peptide SCT proteins. The resulting AAPC-beads were washed twice with PBS, stored in sterile 0.1 M PBS at 4 °C. For phenotype analyses, the AAPC-beads were stained with PE-labeled mouse anti-human HLA-ABC mAb (W6/32) or rat anti-mouse IgG1 for 30 min at 4 °C followed by flow cytometry.

### Enumeration and reactivity evaluation of HBc_18–27_/HBs_183–191_-specific CD8^+^ T cells by AAPC-microplate

Twenty-five adult inpatients with chronic Hepatitis B in the Division of Infectious Diseases, Nanjing Second Hospital Affiliated to Southeast University enrolled in this study with the characteristics: 20 males and 5 females; age: 40.2 ± 10.0 years; 10 subjects were HLA-A2 positive, the others were HLA-A2 negative; alanine aminotransferase: 193.77 ± 77.92 IU L^−1^; aspartate aminotransferase: 116.4 ± 104.91 IU L^−1^; total bilirubin: 20.83 ± 6.71 umol L^−1^; HBV-DNA: > 1000 copies mL^−1^. In addition, fifteen HLA-A2-positive healthy donors also enrolled in this research, who participated in routine health examination at the Department of Laboratory Medicine, Nanjing KingMed Diagnostics Co. Ltd. (an independent clinical laboratory). The research was carried out according to The Code of Ethics of the World Medical Association (Declaration of Helsinki, 1964), and informed consent was obtained from each patient and donor. The protocol was approved by the Ethics Committee of Nanjing Second Hospital Affiliated to Southeast University (Institutional Review Board). Each HLA-A2-positive subject, detected by PE-labeled anti-HLA-A2 mAb staining plus flow cytometry, was further genotyped for HLA-A2 allele using polymerase chain reaction with sequence-based genotyping (PCR-SBT) as recommended by International Histocompatibility Working Group (IHWG). Heparinized blood samples from all subjects were collected and fleshly used for the detection of AST cells in the 96-well AAPC-microplate.

Firstly, 3 × 10^6^ of peripheral blood mononuclear cells (PBMC) were prepared from each blood sample by density-gradient centrifugation, blocked with human Fc receptors blocking antibodies, and seeded into three replicate wells in a 96-well round-bottom plate (1 × 10^6^ cells well^−1^). Then, CD8^+^ T cells were separated using a Dynabeads untouched human CD8^+^ T cell negative selection kit as described, and transferred into a transparent 96-well flat-bottom plate which pre-coated with IFN-γ-capturing antibody. Secondly, five types of HBc_18–27_ or HBs_183–191_-specific AAPC-beads were mixed together and seeded into each well containing CD8^+^ T cells and co-incubated for 2 hrs on a mild shaker at RT. The ratio of each type of AAPC-beads to PBMCs was 1:1. Then, cells were sorted on microplate Magnet as described. The cells and AAPC-beads retaining in each well were maintained in serum-free cell culture medium and further incubated for 24 hrs in a humidified incubator with 5% CO_2_ at 37 °C. Consequently, the cells in each well were counted under microscope or using the automated cell counter as described. The frequency of cells sorted by AAPC-beads in PBMC population was calculated as a mean value of three replicate wells. After cell counting, cells and AAPC-beads were discarded by PBS washing. Finally, IFN-γ spots were developed and counted as described. The percentage of IFN-γ-secreting cells in the HBc_18–27_/HBs_183–191_-specific CD8^+^ T cell population was calculated as a mean value of three replicate wells.

### Enumeration of HBc_18–27_/HBs_183–191_-specific CD8^+^ T cells by traditional flow cytometry

HLA-A2-Ig dimer was loaded with HBc_18–27_ and HBs_183–191_ peptides, respectively, according to the manufacturer’s protocol. The PBMCs from each blood sample were first blocked with human Fc receptors blocking antibodies for 30 min and then incubated with the mixture (1:1:1) of HLA-A2-Ig/HBc_18–27_ dimer, HLA-A2-Ig/HBs_183–191_ dimer, and APC-labeled anti-mouse IgG1 for 1 hr at 4 °C. After washing, FITC-anti-human CD8 and PE-anti-human CD3 were added for additional 30-min incubation followed by flow cytometry. The frequency of APC^+^/FITC^+^ cells in PBMC population was calculated.

### Statistics

Statistical analyses were performed using GraphPad Prism 6.0 (GraphPad, La Jolla, CA) or SPSS 18.0 (SPSS, Chicago, Illinois, USA) software. Paired, two-tailed Student’s t-test was used to determine significant differences between methodological groups. *P* value less than 0.05 was considered significant difference. The correlation coefficient of AAPC-microplate method and traditional methods was analyzed by two-tailed Pearson.

## Electronic supplementary material

Supplementary Tables and Figures
