# Supplementary material for: Frequency and reactivity of antigen-specific T cells were concurrently measured through the combination of artificial antigen-presenting cell, MACS and ELISPOT
Source: Sci Rep. 2017 Nov 27;7:16400. doi: 10.1038/s41598-017-16549-1 (PMC5703716; doi:10.1038/s41598-017-16549-1)
Supplement: Supplementary file 1 — Supplementary Tables and Figures [file 41598_2017_16549_MOESM1_ESM.pdf]

Frequency and reactivity of antigen-specific T cells were concurrently measured through the combination of artificial antigen-presenting cell, MACS and ELISPOT

Chuanlai Shen[1, †, *], Tao Xu[1, †], You Wu[1], Xiaoe Li[1], Lingzhi Xia[2], Wei Wang[1], Khawar Ali Shahzad[1], Lei Zhang[1], Xin Wan[1], Jie Qiu[3]

[1]Department of Microbiology and Immunology, Southeast University Medical School, Nanjing, Jiangsu, China; [2]Department of Laboratory Medicine, Nanjing KingMed Diagnostics Company Limited, Nanjing, Jiangsu, China; [3]Division of Infectious Diseases, Second Hospital of Nanjing, Affiliated Second Hospital of Southeast University, Nanjing, Jiangsu, China

[†]These authors contributed equally to this work.

*Corresponding authors：Jie Qiu and Chuanlai Shen

Jie Qiu, M.D., Division of Infectious Diseases, Affiliated Second Hospital of Southeast University, Nanjing, Jiangsu, China; E-mail: 13851507229@163.com

Chuanlai Shen, Ph.D., Professor, Department of Microbiology and Immunology, Medical School, Southeast University, 87 Dingjiaqiao Rd, Nanjing, Jiangsu Province 210009, China

Fax: +86-2583324887; Telephone: +86-2583272454; E-mail: chuanlaishen@seu.edu.cn

Table S1 Percentages of $OVA_{257\text{-}264}$-specific $CD8^+$ T cells in the lymphocyte population of OT-1 mice detected by two methods.

OT-1 mouse No	$H\text{-}2K^b$/OVA dimer staining +FACS	AAPC-microplate (corrected) †	AAPC-microplate (mean ± SD)	AAPC-microplat e(CV)
1	11.82%	11.27%	10.39 ± 1.26%	12.06%
2	15.27%	14.77%	14.67 ± 0.75%	5.10%
3	10.13%	9.40%	9.09 ± 0.73%	8.11%
4	15.78%	12.62%	12.18 ± 0.53%	4.42%
5	21.45%	21.85%	19.37 ± 2.04%	10.50%
6	8.55%	9.05%	8.94 ± 0.23%	4.40%
7	10.10%	10.73%	10.40 ± 1.01%	8.10%
8	8.85%	9.00%	8.53 ± 0.41%	5.12%
9	21.69%	21.97%	23.01 ± 1.48%	6.44%
10	25.86%	25.16%	26.00 ± 1.04%	4.01%
11	21.76%	20.20%	18.87 ± 1.19%	6.31%
12	23.34%	22.20%	22.78 ± 2.07%	9.08%
13	20.39%	19.05%	19.17 ± 1.27%	6.63%
14	26.04%	25.51%	25.05 ± 1.06%	4.24%
15	26.08%	26.79%	26.44 ± 0.79%	3.11%

† The frequency detected by AAPC-microplate was corrected by linear regression equation for each sample.

Table S2 Methodological reproducibility of AAPC-microplate method.

OT-1 mouse No	AAPC-microplate	positive cells (Mean ± SEM)	positive cells (corrected)[†]	R^2 value	Within-run CV	Between-run CV
No.3	First test	10.40 ± 0.53%	9.50%	0.9980	5.18%	
	Second test	10.05 ± 0.68%	9.12%	0.9970	6.70%	6.88%
	Third test	9.09 ± 0.73%	9.40%	0.9961	8.11%	
No.13	First test	19.17 ± 1.27%	19.05%	0.9976	6.63%	
	Second test	19.06 ± 0.78%	18.94%	0.9975	4.12%	3.22%
	Third test	18.16 ± 2.02%	17.96%	0.9876	11.14%	
No.15	First test	25.44 ± 0.79%	25.47%	0.9991	3.11%	
	Second test	24.80 ± 1.09%	25.59%	0.9993	4.41%	2.40%
	Third test	23.17 ± 2.46%	24.49%	0.9975	10.62%	

[†] The frequency detected by AAPC-microplate was corrected by linear regression equation for each sample.

Table S3 Reactivity of $OVA_{257-264}$-specific $CD8^+$ T cells detected by AAPC-micropplate.

OT-1 mouse No	H-$2K^b$/OVA dimer staining +FACS	AAPC-microplate (enumeration)†	Traditional ELISPOT	AAPC-microplate (Reactivity)†
4	15.78%	12.62%	7.08%	47.12%
5	21.45%	21.85%	7.97%	47.59%
6	8.55%	9.05%	4.08%	48.75%
7	10.10%	10.73%	5.02%	47.48%
8	8.85%	9.00%	4.65%	49.64%
11	21.76%	20.20%	10.44%	43.83%
12	23.34%	22.20%	9.81%	41.87%
14	26.04%	25.51%	14.98%	48.99%

† The frequency detected by AAPC-microplate was corrected by linear regression equation for each sample.

Table S4 Percentages of HBc_{18-27}/$HBs_{183-191}$-specific $CD8^+$ T cells in the PBMCs from all subjects as detected by the AAPC-microplate method and traditional flow cytometry.

**Sample No.**	**HLA-A2$^+$ patients**		**HLA-A2$^-$ patients**		**HLA-A2$^+$ health donors**	
	**AAPC-microplate**	**FACS**	**AAPC-microplate**	**FACS**	**AAPC-microplate**	**FACS**
No. 1	0.282%	0.25%	0.025%	0.03%	0.025%	0.03%
No. 2	0.135%	0.11%	0.021%	0.01%	0.022%	0.02%
No. 3	0.357%	0.37%	0.032%	0.02%	0.017%	0.01%
No. 4	0.126%	0.11%	0.024%	0.01%	0.015%	0.01%
No. 5	0.191%	0.17%	0.017%	0.01%	0.021%	0.01%
No. 6	0.213%	0.19%	0.026%	0.03%	0.026%	0.03%
No. 7	0.285%	0.30%	0.018%	0.02%	0.015%	0.01%
No. 8	0.134%	0.10%	0.022%	0.01%	0.014%	0.01%
No. 9	0.257%	0.28%	0.015%	0.02%	0.016%	0.01%
No. 10	0.219%	0.18%	0.043%	0.03%	0.023%	0.01%
No. 11			0.028%	0.03%	0.027%	0.03%
No. 12			0.016%	0.01%	0.023%	0.01%
No. 13			0.023%	0.01%	0.018%	0.01%
No. 14			0.035%	0.04%	0.036%	0.04%
No. 15			0.029%	0.03%	0.024%	0.03%

No statistical difference was found between the two methods as analyzed by paired, two-tailed Student’s t-tests. $p > 0.05$.

Supplementary Figure 1:

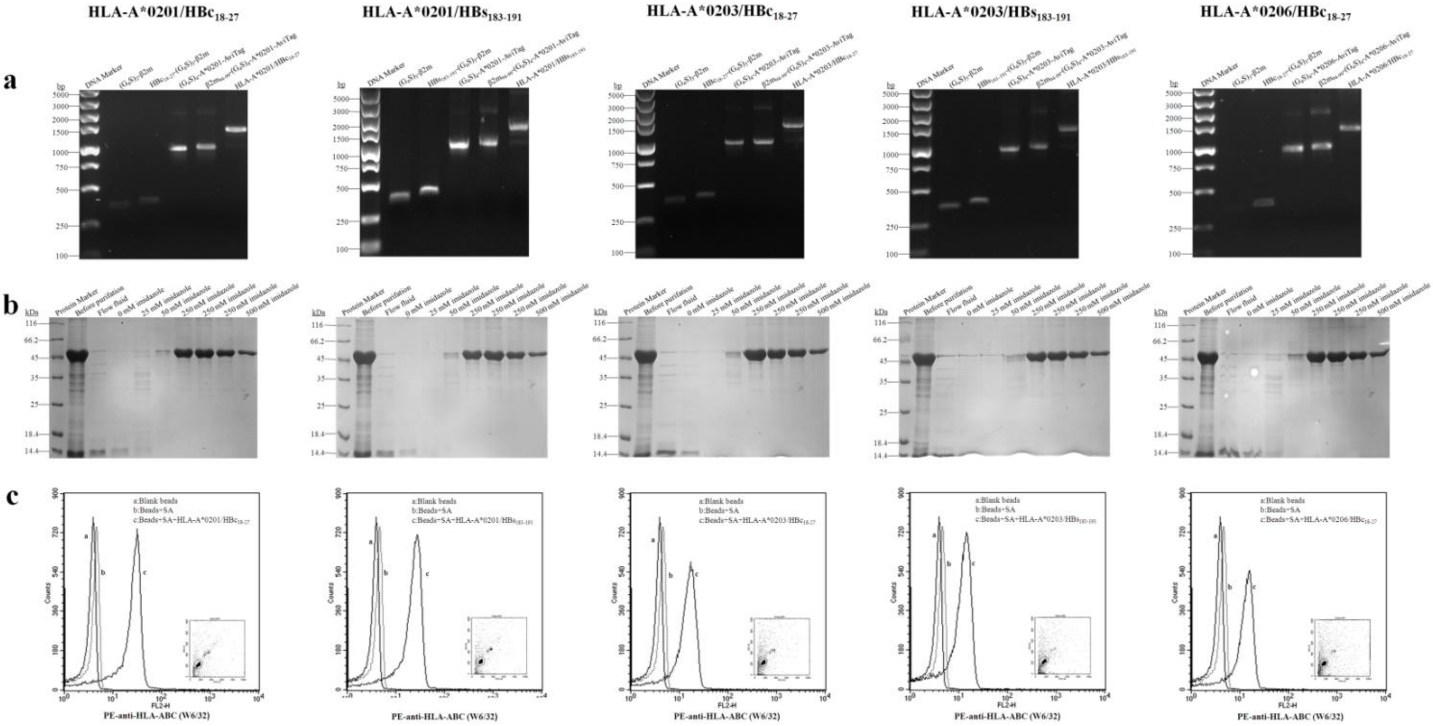

Figure S1 Construction, expression and phenotype analysis of 5 types of HLA-A2/HBV peptide complexes. The recombinant SCT genes of HLA-A*0201/HBc$_{18-27}$, HLA-A*0201/HBs$_{183-191}$, HLA-A*0203/HBc$_{18-27}$, HLA-A*0203/HBs$_{183-191}$, and HLA-A*0206/HBc$_{18-27}$ complexes were successfully constructed and inserted into plasmid pET28a by overlap extension PCR and One-step cloning. **Fig. S1a** reveals the electrophoresis of five DNA fragments amplified during overlap extension PCR. The SCT proteins were expressed in *Escherichia coli* followed by collection and purification. **Fig. S1b** displays the purification of inclusion body by Ni^{+}-chelating affinity column. Then, the SCT proteins were refolded, concentrated, biotinylated, and immobilized onto the cell-sized magnetic beads indirectly by streptavidin. **Fig. S1c** shows that each type of AAPC-beads can significantly bind with PE-labeled anti-human HLA-ABC (W6/32), a conformation-specific mAb, implying the correct conformational structure and appropriate orientation of HLA-A2/peptide multimers onto beads.

Supplementary Figure 2:

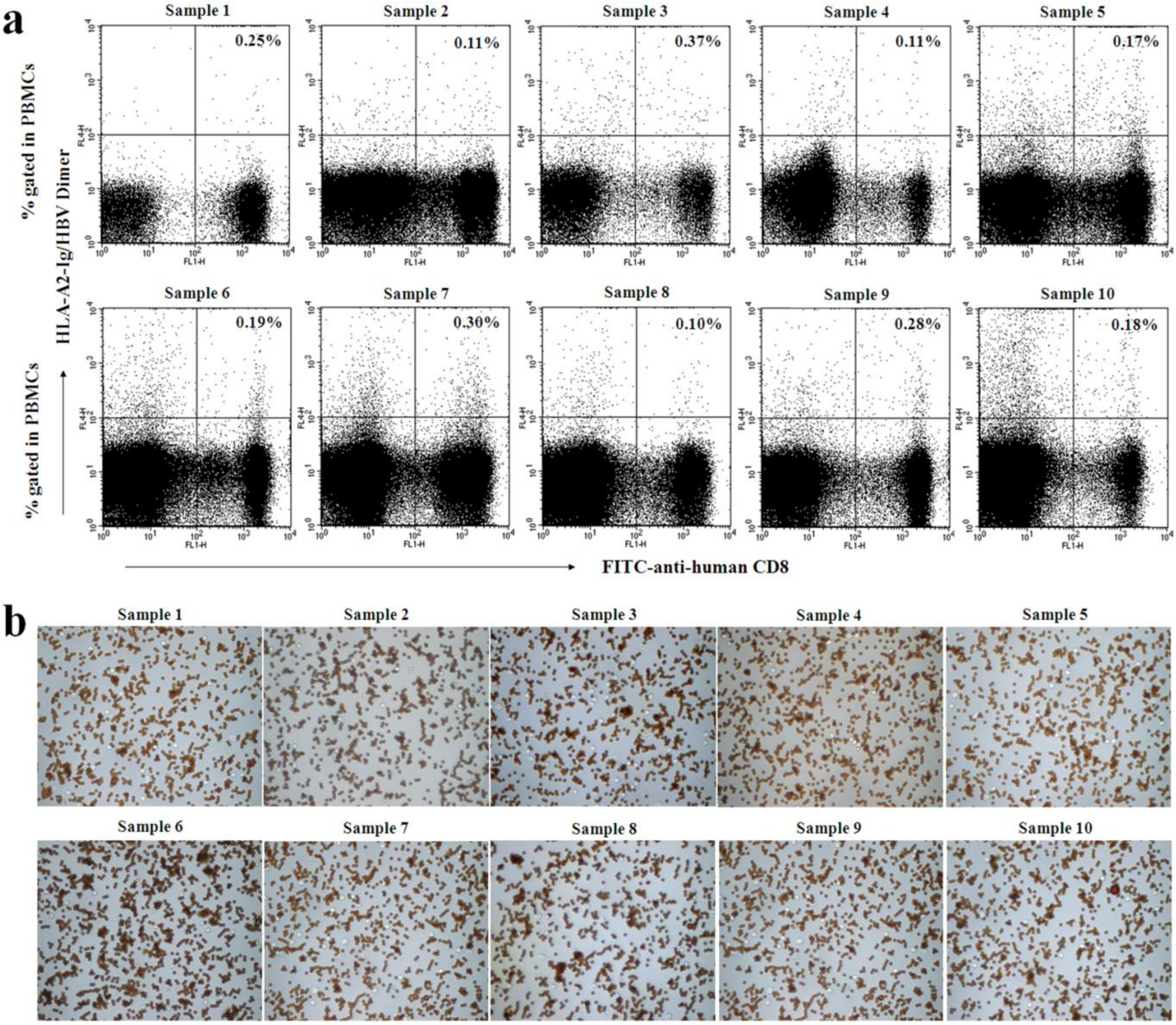

Figure S2 Enumeration of HBc$_{18-27}$/HBs$_{183-191}$-specific CD8^{+} T cells in the PBMCs from 10 HLA-A2-positive patients with chronic Hepatitis B by the AAPC-microplate method and traditional flow cytometry. **(a)** The flow cytometric dot spots for each HLA-A2-positive patient. **(b)** AAPC-bead sorting picture for each HLA-A2-positive patient.

Supplementary Figure 3:

a

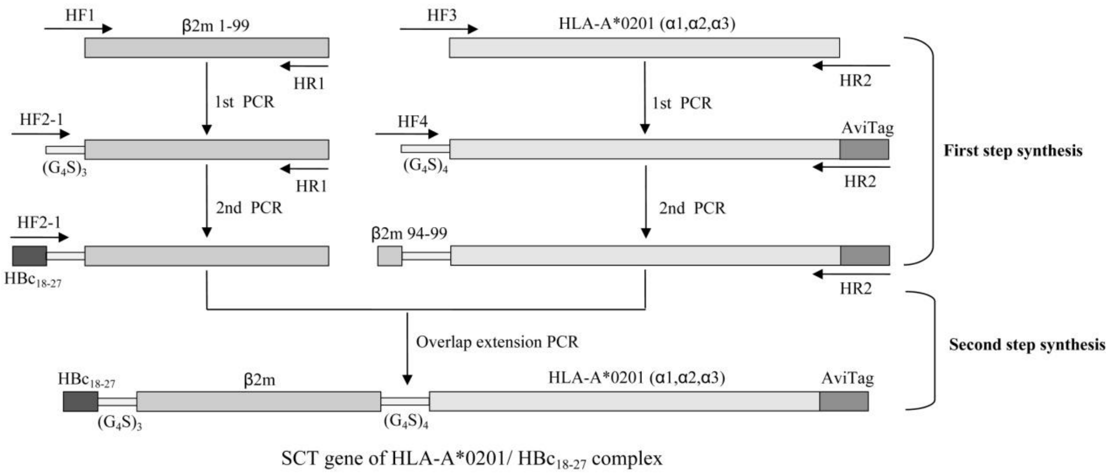

b

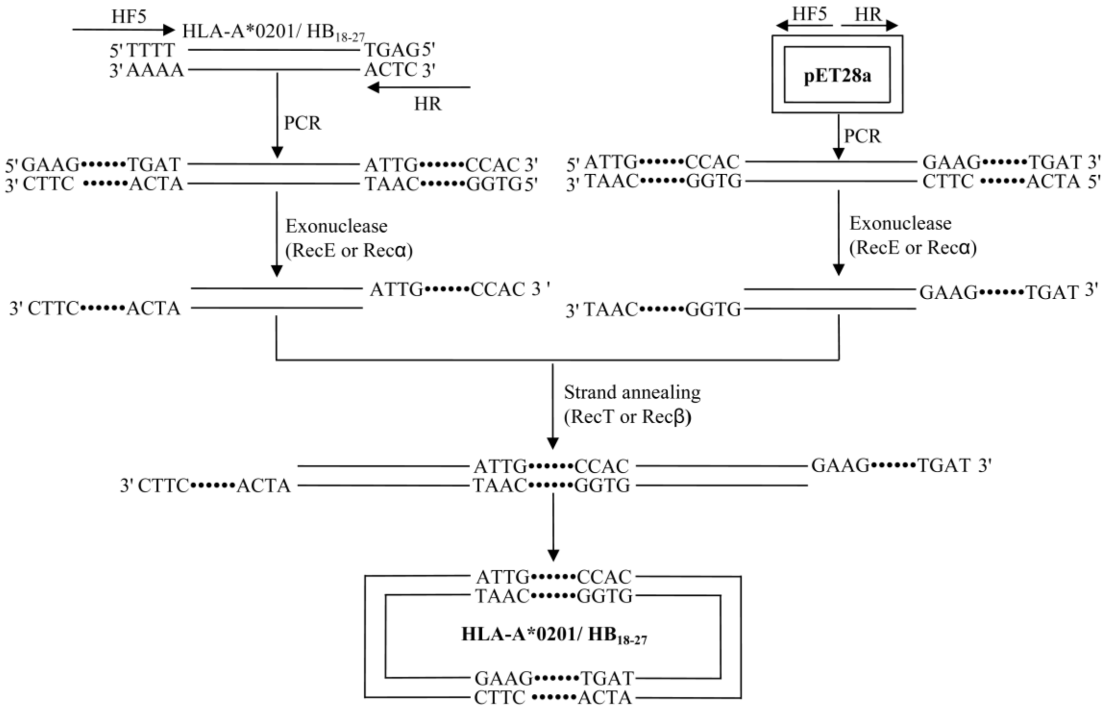

Figure S3 Construction of recombinant plasmid pET28a-HLA-A*0201/HBc_{18-27}. **(a)** Schematic representation of overlap extension PCR for the splicing of HLA-A*0201/HBc_{18-27} single-chain fusion gene. **(b)** Schematic representation of One-step cloning technique for the construction of recombinant plasmid pET28a-HLA-A*0201/HBc_{18-27}.

Below is method to generate the recombinant plasmid pET28a-HLA-A*0201/HBc_{18-27}.

1. Construction of single-chain-trimer (SCT) gene for HLA-A*0201/HBc_{18-27} complex

A two-step strategy combing the assembly PCR and overlap extension PCR process was developed to synthesize the SCT gene of HLA-A*0201/HBc_{18-27} complex (**Fig. S3a**). First, the recombinant plasmid pET28-β2m containing the human β2m cDNA was used as a template to construct the HBc_{18-27}-$(G_4S)_3$-β2m fusion gene with two sequential PCR using the primer combinations HF1/HR1 first and HF2-1/HR1 later. Then, the recombinant plasmid pET28-HLA-A*0201 containing HLA-A*0201 (α1, α2, α3) cDNA was used as a template to amplify the fusion gene consisting of $β2m_{94-99}$, $(G_4S)_4$, HLA-A*0201, and AviTag from the 5' end to the 3' end. Two-round PCR was performed using the primer combinations HF3/HR2 first and HF4/PR2 later. The resulting PCR product was finally fused with the $OVA_{257-264}$-linker1-β2m fusion gene by overlap extension PCR with the primer combination of HF5/HR.

2. Construction of recombinant plasmid pET28a- HLA-A*0201/HBc_{18-27}

As shown in **Fig. S3b**, the One-step cloning technique was used for cloning of HLA-A*0201/HBc_{18-27} SCT fusion gene in pET28a vector. A linear vector was amplified by PCR with the HF5 and HR primers from pET28a. Also, the SCT gene of HLA-A*0201/HBc_{18-27} complex was amplified using HF5 and HR primers. Primer HF5 contains 17nts of pET28a and 15nts of the SCT gene. Primer HR contains 15nts of pET28a and 15nts of the SCT gene. Then, the PCR product of SCT gene was recombinated with the linear pET28a vector using the Exnase^{TM} II of ClonExpress^{TM} II One Step Cloning Kit (Vazyme, China), an Exnase consisting of RecE/Redα, RecT/Redβ and Recγ protein. RecE/Redα can bind to the ends of dsDNA fragments and processively degrade the linear dsDNA in a 5' to 3' direction; RecT/Redβ can bind to ssDNA and promote strand annealing;

Redγ is a DNA mimic that inhibits the *E.coli* RecBCD exonuclease and prevents degradation of linear dsDNA. Briefly, the purified PCR product of SCT gene (60 ng) was mixed with 100 ng of the linear vector, 2 μl of Exnase^{TM} II and 4μl of 5×CE II buffer. The mixture was incubated at 37 ^{o}C for 30 min followed by 5 min on ice, and then transformed into *E. coli* DH5α (Sangon, China). The plasmid encoding the SCT molecule with a C terminal biotin protein ligase BirA substrate peptide (AviTag) and a His6 tag was constructed. The positive clones containing the recombinant plasmid HLA-A*0201/HBc$_{18-27}$ were screened by colony PCR using primers T7 and T7 ter, and were confirmed by nucleotide sequencing.

Table S5 Primers used for the construction of SCT gene

Name	Sequence (5′-3′)
HF1	GGAGGTGGCGCTTCAGGAGGTGGCGGTTCAGGAGGTGGC GGTTCAGATATCCAGCGTACTCCAAAGATT
HF2-1	TTTTTACCTTCTGATTTCTTTCCTTCGGTC GGAGGTGGCGCTTCA
HF2-2	TTCTTGTTGACAAGAATCCTCACAATA GGAGGTGGCGCTTCA
HF3	GGAGGTGGCGGTTCAGGAGGTGGCGGTTCAGGAGGTGGCG GTTCAGGAGGTGGCGGTTCAGGCTCCCACTCCATGAGGTA
HF4	AAGTGGGATCGAGACATGGGAGGTGGCGGTTCAGGAGGTG GCGGTTCAGGAGGTGGCGGTTCAGGAGGTGGCGGTTCAGG
HR1	CATGTCTCGATCCCACTT
HR2	CTCATGCCATTCAATTTTCTGTGCTTCGAAAATATCATTCAA GCCGCCCCCGGTGAGGGGCTTGGGCAGAC
HF5	GAAGGAGATATACCATGTTTTTACCTTCTGAT
HF6	GAAGGAGATATACCATGTTCTTGTTGACAAGA
HR	GTGGTGGTGGTGGTGCTCATGCCATTCAAT

Table S6 Primer pairs used to construct the HLA-A2/HBV peptide SCT genes

	Gene	Template	5'primer	3' primer	Length (bp)
HLA-A*0201/HBc$_{18-27}$	HBc$_{18-27}$-(G$_4$S)$_3$-β2m	β2m	HF1	HR1	342bp
		(G$_4$S)$_3$-β2m	HF2-1	HR1	372bp
	β2m$_{94-99}$-(G$_4$S)$_4$-HLA-A*0201-AviTag	HLA-A*0201	HF3	HR2	915bp
		(G$_4$S)$_4$-HLA-A*0201-AviTag	HF4	HR2	933bp
	HLA-A*0201/ HBc$_{18-27}$	HBc$_{18-27}$-(G$_4$S)$_3$-β2m β2m$_{94-99}$-(G$_4$S)$_4$-HLA-A*0201-AviTag	HF2-1	HR2	1287bp
HLA-A*0201/HBs$_{183-191}$	HBs$_{183-191}$-(G$_4$S)$_3$-β2m	(G$_4$S)$_3$-β2m	HF2-2	HR1	369bp
	HLA-A*0201/HBs$_{183-191}$	HBs$_{183-191}$-(G$_4$S)$_3$-β2m β2m$_{94-99}$-(G$_4$S)$_4$-HLA-A*0201-AviTag	HF2-2	HR2	1284bp
HLA-A*0203/HBc$_{18-27}$	β2m$_{94-99}$-(G$_4$S)$_4$-HLA-A*0201-AviTag	HLA-A*0203	HF3	HR2	915bp
		(G$_4$S)$_4$-HLA-A*0203-AviTag	HF4	HR2	933bp
	HLA-A*0203/HBc$_{18-27}$	HBc$_{18-27}$-(G$_4$S)$_3$-β2m β2m$_{94-99}$-(G$_4$S)$_4$-HLA-A*0203-AviTag	HF2-1	HR2	1287bp
HLA-A*0203/HBs$_{183-191}$	HLA-A*0203/HBs$_{183-191}$	HBs$_{183-191}$-(G$_4$S)$_3$-β2m β2m$_{94-99}$-(G$_4$S)$_4$-HLA-A*0203-AviTag	HF2-2	HR2	1284bp
HLA-A*0206/HBc$_{18-27}$	(G$_4$S)$_4$-HLA-A*0206-AviTag	HLA-A*0206	HF3	HR2	915bp
		(G$_4$S)$_4$-HLA-A*0206-AviTag	HF4	HR2	933bp
	HLA-A*0206/HBc$_{18-27}$	HBc$_{18-27}$-(G$_4$S)$_3$-β2m β2m$_{94-99}$-(G$_4$S)$_4$-HLA-A*0206-AviTag	HF2-1	HR2	1287bp
